# NONcNZO10/LtJ and TALLY‐HO/JngJ Mice as Novel Models of Diabetic Peripheral Neuropathy

**DOI:** 10.1096/fj.202600848R

**Published:** 2026-09-05

**Authors:** Stéphanie A. Eid, Andrew D. Carter, Emily J. Koubek, John M. Hayes, Diana M. Rigan, Chloe Kiriluk, Crystal M. Pacut, Phillipe D. O'Brien, Jihyun Park, Dae‐Gyu Jang, Eva L. Feldman

**Affiliations:** ^1^ Department of Neurology University of Michigan Ann Arbor Michigan USA; ^2^ Department of Anatomy, Cell Biology and Physiological Sciences American University of Beirut Faculty of Medicine and Medical Center, Beirut Lebanon

**Keywords:** caloric restriction, diabetic peripheral neuropathy, disease models, NONcNZO10/LtJ, polygenic, TALLYHO/JngJ

## Abstract

Diabetic peripheral neuropathy (DPN), a severe complication of Type 2 diabetes (T2D), is marked by progressive distal‐to‐proximal axonal degeneration. Although animal models have advanced our understanding of DPN pathogenesis, preclinical successes rarely translate into effective therapies, underscoring the need for models that more faithfully mirror human disease. In this study, we characterized two polygenic mouse strains, NONcNZO10/LtJ (RCS10) and TALLYHO/JngJ (TH), and found that both models developed obesity, hyperglycemia, dyslipidemia, and DPN by 24 weeks of age. However, hyperinsulinemia was observed only in RCS10 mice. Compared to monogenic and high‐fat diet‐induced models, the metabolic and neuropathic phenotypes of RCS10 and TH more closely resembled those of human DPN. In the second phase of the study, RCS10 mice were subjected to a calorie‐restricted diet (60% of standard intake) for 8 weeks, which improved metabolic health and restored large fiber function, although intraepidermal nerve fiber density remained unchanged. Together, these findings identify RCS10 and TH mice as clinically relevant polygenic models for studying DPN pathogenesis and evaluating targeted therapeutic strategies.

## Graphical abstract

Animal models of diabetic peripheral neuropathy (DPN) are essential for understanding disease mechanisms and evaluating therapies. We characterized two polygenic mouse strains, RCS10 and TH, and found that both developed obesity, hyperglycemia, dyslipidemia, and DPN. Compared with monogenic and high‐fat diet‐induced models, their metabolic and neuropathic phenotypes more closely resembled human DPN. Calorie restriction in RCS10 mice improved metabolic health and restored large‐fiber function. These findings establish RCS10 and TH as clinically relevant models for studying DPN pathogenesis and therapies.

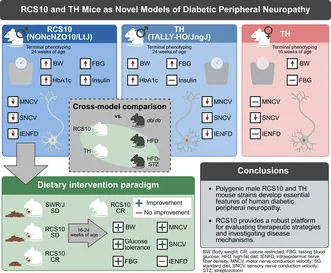

## 1 Introduction

Type 2 diabetes (T2D) is increasingly prevalent, reaching epidemic proportions across the globe [1]. One of its most common and debilitating complications is diabetic peripheral neuropathy (DPN), affecting approximately 50% of individuals over the course of the disease [2]. DPN is a progressive condition, characterized by distal‐to‐proximal peripheral nerve damage, often leading to neuropathic pain, gait and stability problems, and, in severe cases, lower limb amputation [3, 4]. Despite its significant prevalence and clinical burden, there are no effective disease‐modifying therapies for DPN, highlighting the urgent need for a better understanding of its pathogenesis.

Mouse models of T2D have been instrumental in advancing our understanding of DPN pathogenesis [5, 6]. Common monogenic models, such as the leptin‐deficient *ob/ob* and leptin receptor‐mutant *db/db* mice, develop hallmark features of T2D, including obesity, insulin resistance, hyperglycemia, and neuropathic changes [5, 7, 8]. However, these mice undergo rapid disease progression, with severe hyperglycemia, overt hyperphagia, and intervention‐resistant DPN, features that differ from the gradual, progressive onset typical of human T2D DPN [9, 10]. Furthermore, loss of leptin signaling in these models can independently disrupt glucose and lipid metabolism, which may limit the translatability of findings to humans, as some effects may stem from leptin deficiency rather than diabetes alone [11].

As an alternative to monogenic models, diet‐induced models of T2D use high‐fat diet (HFD) feeding to induce progressive obesity, insulin resistance, dyslipidemia, and impaired glucose tolerance, closely mimicking the metabolic profile of early or moderate human T2D without overt hyperglycemia. These models also exhibit key features of human DPN, such as impaired sensory responses, delayed nerve conduction velocities (NCVs), and decreased intraepidermal nerve fiber densities (IENFDs) [5, 12, 13]. However, the extent of nerve damage varies widely depending on factors like mouse strain, age, duration of HFD, and the composition of dietary fat, which can limit the reproducibility and translational relevance [5, 14, 15]. While both monogenic and HFD‐induced models have advanced our understanding of DPN pathogenesis, promising preclinical results have yet to translate into effective treatments for patients.

Polygenic mouse models of T2D offer promising alternatives that address many limitations of the monogenic and diet‐induced models of T2D. The NONcNZO10/LtJ (RCS10) mouse is a recombinant congenic strain created by transferring six diabetes‐ and obesity‐linked genetic regions from the NZO/HlLt strain into the nonobese and diabetes‐resistant NON/Lt background [16]. RCS10 mice develop maturity‐onset hyperglycemia, insulin resistance, dyslipidemia, and moderate liver steatosis, but display only moderate obesity and lack confounding traits such as hypercortisolism, hyperphagia, or thermoregulatory defects seen in monogenic models [16, 17]. Another notable polygenic model is the TALLYHO/JngJ (TH) mouse, derived from selective breeding of outbred Theiler Original male mice exhibiting polyuria and glucosuria, followed by intercrossing, backcrossing for diabetic traits, and inbreeding to create an inbred line [18]. Like RCS10, TH mice spontaneously develop hyperglycemia, hyperinsulinemia, hyperlipidemia, and moderate obesity, with a gradual disease progression and absence of overt diabetes compared to monogenic models [19]. Importantly, both RCS10 and TH mice incorporate complex genetic factors underlying diabetes, making them valuable for studying the genetic contributions to the disease, an advantage over diet‐induced models. However, DPN phenotypes in these polygenic strains remain poorly characterized, limiting conclusions about their suitability as models for DPN.

To address this knowledge gap, we first conducted metabolic and DPN phenotyping of the RCS10 and TH mice to evaluate their suitability as DPN models and identify the superior model. Next, using the more robust model, we investigated whether a caloric restriction regimen could reverse nerve dysfunction, thereby demonstrating the potential utility for testing therapeutic interventions for DPN in this model.

## 2 Materials and Methods

### 2.1 Animals

Data were obtained from two cohorts of mice.

Cohort 1 was used to identify the most robust model of DPN and consisted of 4 experimental groups of 10‐week‐old male mice: (1) NONcNZO10/LtJ (RCS10; *n* = 6; strain #004456; RRID:IMSR_JAX:004456; The Jackson Laboratory, Bar Harbor, ME), (2) C57BL/6J (BL6, control for RCS10; *n* = 6; strain #000664; RRID:IMSR_JAX:000664; The Jackson Laboratory), (3) TALLYHO/JngJ (TH; *n* = 9; strain #005314; RRID:IMSR_JAX:005314; The Jackson Laboratory), and (4) SWR/J (Control for TH; *n* = 9; strain #000689; RRID:IMSR_JAX:000689; The Jackson Laboratory) mice. Eight‐week‐old female TH (*n* = 6) and SWR/J (*n* = 6) mice were also purchased from The Jackson Laboratory. Mice were fed *ad libitum* a standard diet (SD; 57% kcal carbohydrates, 29% kcal protein, 13.6% kcal fat from lard; cat #0001319; LabDiet, St. Louis, MO). Terminal metabolic and DPN phenotyping were performed at 16 weeks of age for female TH and SWR/J mice and at 24 weeks of age for male RCS, BL6, TH, and SWR/J mice.

Cohort 2 was used to assess the neuroprotective effects of a dietary intervention paradigm and consisted of 9‐week‐old male RCS10 (*n* = 14) and SWR/J (control for RCS10; *n* = 4) mice fed *ad libitum* a SD. On average, RCS10 mice consumed 5 g chow/day. At 16 weeks of age, half of the RCS10 mice (*n* = 7) were calorically restricted to 60% of their standard intake (3 g chow/day). Baseline metabolic and DPN phenotyping were performed at 16 weeks of age, prior to caloric restriction and terminal phenotyping was performed at 24 weeks of age.

Mice were maintained in a pathogen‐free environment (20°C ± 2°C, 12:12 h light: dark cycle) and water was provided *ad libitum*. Animals were assigned a unique ID and phenotyping was performed in a blinded manner. All protocols and procedures were approved by the University of Michigan Committee on the Use and Care of Animals (Protocol #PRO00008116) and complied with the US National Research Council's Guide for the Care and Use of Laboratory Animals, the US Public Health Service's Policy on Humane Care and Use of Laboratory Animals, and Guide for the Care and Use of Laboratory Animals.

### 2.2 Metabolic Phenotyping

For both Cohorts, body weight and fasting blood glucose (FBG; 4 h fast) were collected every 2 weeks until study termination (24 weeks of age). FBG was measured using an AlphaTrak glucometer (Abbott Laboratories, Abbott Park, IL, USA). Terminal HbA1c levels were quantified by ELISA according to the manufacturer's protocol (Mouse hemoglobin A1C assay kit, cat #80310; Cystal Chem Inc., Elk Grove Village, IL, USA). Plasma insulin and lipid levels were measured at study termination by the University of Michigan Mouse Metabolic Phenotyping Core (MMPC) and the University of Cincinnati MMPC, respectively. For Cohort 2, glucose tolerance testing (GTT) was performed at baseline (16 weeks of age and prior to dietary intervention) and study termination (24 weeks of age), per our published protocols [13, 20]. Briefly, mice were fasted for 4 h and then administered a glucose bolus (1 g/kg body weight) via intraperitoneal injection. Blood glucose levels were measured from tail vein blood at baseline (0 min) and at 15, 30, 60, and 120 min post‐injection using an AlphaTrack glucometer. Body composition of Cohort 2 was determined at study termination by the University of Michigan MMPC, as previously reported [21, 22, 23].

### 2.3 DPN Phenotyping

Baseline (16 weeks of age) motor and sensory NCVs (Cohort 2) and terminal (24 weeks of age) NCVs and IENFDs (Cohorts 1 and 2) were quantified per our published protocols [24, 25]. Nerve conduction studies measured large fiber function and were performed under anesthesia with isoflurane while maintaining body temperature at 34°C with a heating lamp. For sensory NCVs, a sural nerve was stimulated at the ankle using bipolar electrodes with supramaximal stimulation, and sensory nerve action potentials were recorded from the dorsum of the foot. NCVs were calculated by dividing the distance between the stimulation (ankle) and recording site (foot dorsum) by the onset latency of the sensory nerve action potential. Motor NCVs were measured by applying supramaximal stimulation to the sciatic nerve at two sites: distally at the ankle and then proximally at the sciatic notch, using bipolar electrodes. Compound muscle action potentials were recorded from the dorsum of the foot. The motor NCV was calculated by dividing the distance between the two stimulated sites by the difference in onset latencies of the recorded responses. IENFDs to evaluate small fiber anatomy were assessed by collecting the plantar surface of the hind paw. Tissue samples were fixed in 2% Zamboni Fixative (cat #1459A; Newcomer Supply, Waunakee, WI, USA), washed, embedded, cryoprotected, and sectioned at 30 μm thickness. Sections were immunostained with anti‐protein gene product 9.5 (PGP9.5; cat #14730‐1‐ap; RRID:AB_2210497; used at 1:3000; Proteintech, Rosemont, IL, USA) antibody to visualize the axons. Three z‐series images were obtained using a confocal microscope (Leica SP5; 20 × 1.2 water‐immersion objective; 1024 × 1024 pixel resolution; Leica Microsystems, Wetzlar, Germany). MetaMorph software (v.7.7.0.8; Molecular Devices, San Jose, CA, USA) was used to determine the number of PGP9.5‐positive nerve fibers crossing the dermal‐epidermal junction per millimeter of epidermal length.

### 2.4 Previous Datasets

To compare the two new animal models to established T2D mouse models, we analyzed previously published datasets from our group [20, 22]. Specifically, metabolic and DPN phenotyping were extracted from studies involving 22 weeks old male *db/+* control and *db/db* mice on a C57BLKS/J background (monogenic T2D model) and 36 weeks old C57BL/6J male mice fed either a standard diet (SD), a 60% HFD starting at 5 weeks of age, or a HFD starting at 5 weeks of age with administration of streptozotocin (STZ) at 12 weeks of age (HFD‐STZ, diet‐induced T2D models) [20, 22]. Briefly, for the HFD‐STZ model, mice received two low doses of STZ (75 mg/kg followed 72 h later by 50 mg/kg, intraperitoneally), resulting in diet‐induced insulin resistance accompanied by β‐cell dysfunction, hypoinsulinemia, and hyperglycemia, more closely resembling later‐stage T2D.

### 2.5 Statistical Analysis

Statistical analysis of metabolic and DPN phenotyping was performed using GraphPad Prism (v.10.2.2; RRID:SCR_002798; GraphPad Software Inc., San Diego, CA, USA). No formal statistical methods were used to predetermine sample size; however, the chosen sample size (*n* = 6 mice total) is consistent with prior studies in the field and with our own work, in which similar group sizes were calculated to achieve 90% power and 95% sensitivity to detect statistically significant DPN phenotypes [13, 14, 21, 26]. Comparisons between two groups were analyzed by unpaired *t*‐test. Comparisons across multiple groups were analyzed by one‐way ANOVA followed by Tukey's *post hoc* test. Longitudinal body weight and FBG for Cohort 1 and baseline GTT for Cohort 2 were analyzed by repeat measures two‐way ANOVA followed by Sidak's *post hoc* test. Longitudinal body weight, FBG, and terminal GTT for Cohort 2 were analyzed by repeat measures two‐way ANOVA followed by Tukey's *post hoc* test. To confirm the assumptions underlying parametric tests, we assessed variance using Levene's test for *t*‐tests and ANOVA and likelihood ratio tests (LRTs) for longitudinal analyses, and assessed normality using the Shapiro–Wilk test (Table S1). Sensitivity analyses demonstrated consistent results across statistical approaches (Tables S2–S4).

Comparisons across monogenic (*db/+* control, *db/db*), diet‐induced (SD control, HFD, HFD‐STZ), and polygenic (BL6 control, RCS10; SWR/J control, TH) T2D models were conducted using ANOVA with linear contrasts to assess whether percentage differences in metabolic factors and DPN phenotype (treatment vs. control) varied significantly among the five T2D models. Pearson's correlation coefficient analysis was used to assess pairwise correlations between various metabolic and DPN parameters in monogenic, diet‐induced, and polygenic T2D models. Of note, the correlation analysis for TH mice required combining data from two different data sets. To facilitate this, a statistical matching (or data fusion) method was employed [27]. Data are represented as mean ± standard deviation, with statistical significance set at *p* < 0.05.

## 3 Results

### 3.1 RCS10 And TH Mouse Models Display a T2D Phenotype

**FIGURE 1:**
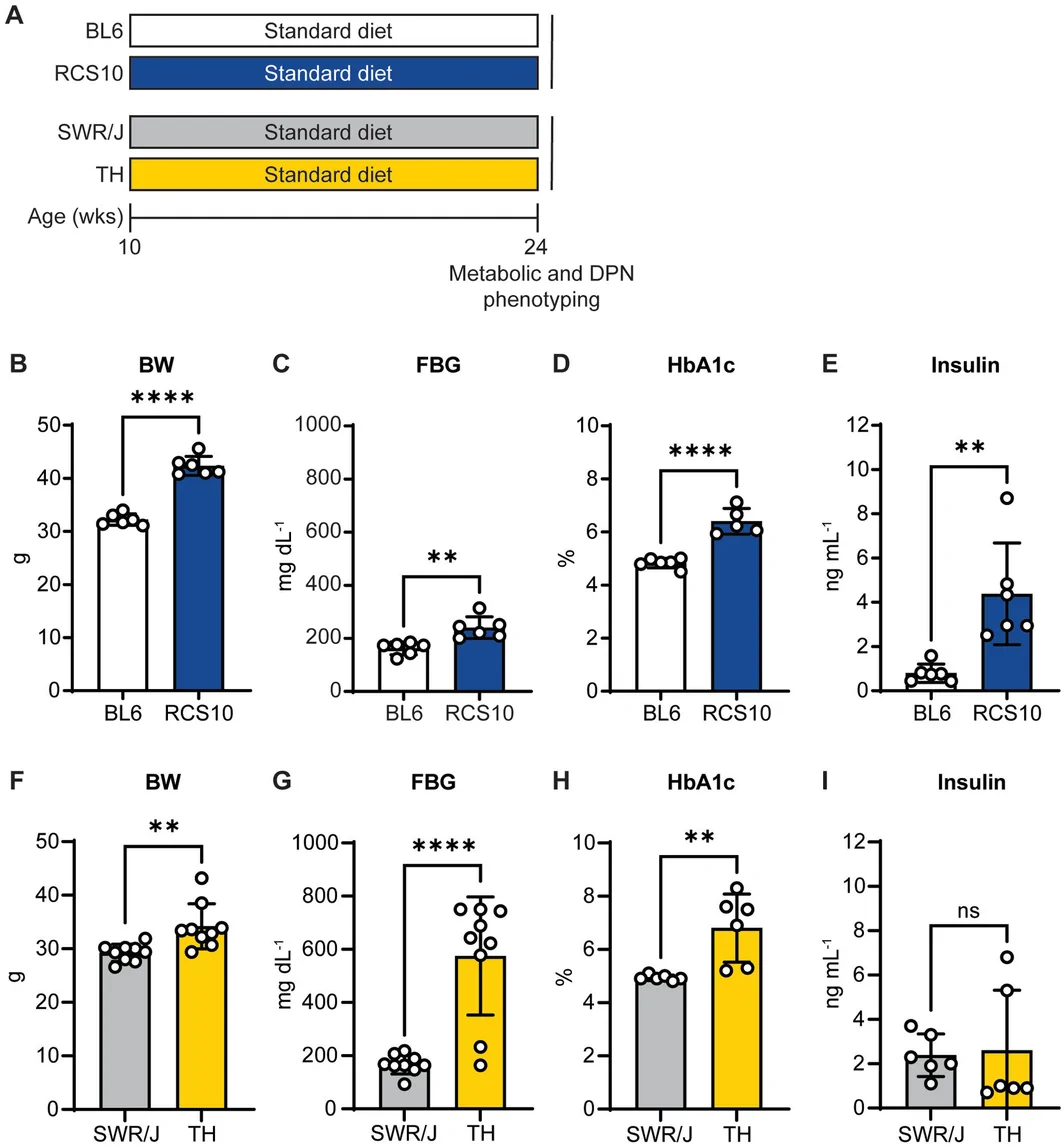
RCS10 and TH mice develop metabolic dysfunction. (A) Study design for model phenotyping. Ten‐week‐old NONcNZO10/LtJ (RCS10), C57BL/6J (BL6, control for RCS10), TALLYHO/JngJ (TH) and SWR/J (control for TH) mice were fed a standard diet and monitored until 24 weeks of age. Body weight (BW) and fasting blood glucose (FBG) were measured biweekly. At 24 weeks of age, terminal assessments included BW, FBG, HbA1c, plasma insulin and lipids, as well as diabetic peripheral neuropathy (DPN) phenotyping (nerve conduction velocities and intraepidermal nerve fiber densities). Terminal (B, F) BW, (C, G) FBG, (D, H) HbA1c, and (E, I) insulin in RCS10 and BL6 mice (B–E, *n* = 6) and TH and SWR/J mice (F, G, *n* = 6; H, I, *n* = 9). Each data point represents an individual animal. Data are represented as mean ± standard deviation. Two‐tailed unpaired *t*‐test. ns, not significant, *p < 0.05, **p < 0.01, ****p < 0.0001.

**FIGURE 2:**
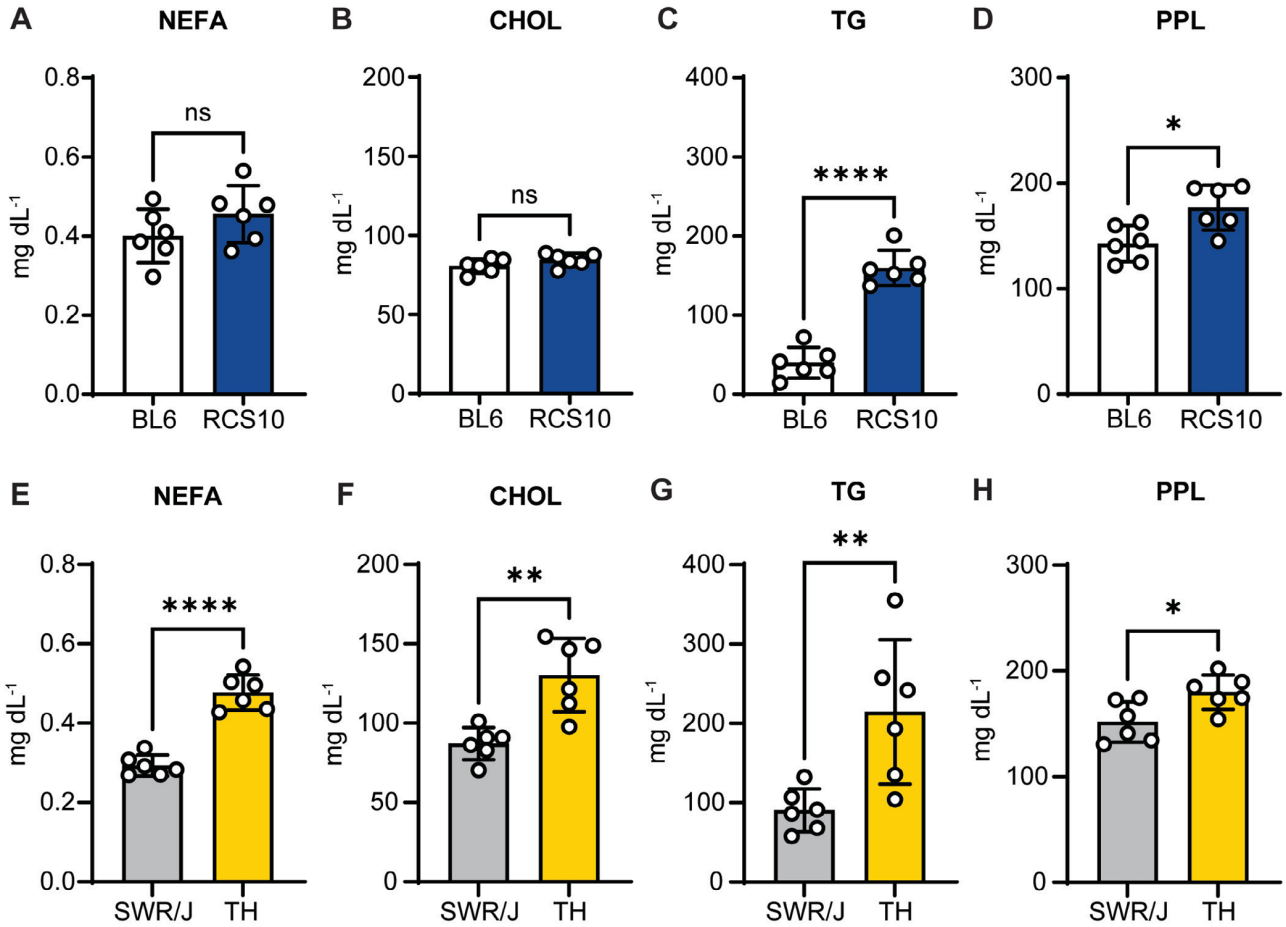
RCS10 and TH mice have altered lipid profiles. Terminal (A, E) non‐esterified fatty acid (NEFA), (B, F) cholesterol (CHOL), (C, G) triglyceride (TG), and (D, H) phospholipid (PPL) levels in BL6 and RCS10 mice (A–D, *n* = 6) and SWR/J and TH mice (E–H, *n* = 6). Each data point represents an individual animal. Data are represented as mean ± standard deviation. Two‐tailed unpaired *t*‐test. ns, not significant, *p < 0.05, **p < 0.01, ****p < 0.0001.

In the first part of our study, we compared the metabolic phenotypes of the RCS10 and TH mouse models (Figure 1A). From 10 to 24 weeks of age, both RCS10 and TH mice gained significantly more weight than their respective controls, with RCS10 mice exhibiting the greatest weight gain (Figure 1B,F; Figure S1A,C). By the end of the study, both models showed elevated FBG and HbA1c levels (Figure 1C,D,G,H; Figure S1B,D). Notably, only RCS10 mice developed significant hyperinsulinemia (*p* = 0.004; Figure 1E,I). At 24 weeks, both models demonstrated features of dyslipidemia, although the profiles differed. RCS10 mice had elevated triglycerides and phospholipids, while TH mice showed increased levels of non‐esterified fatty acids (NEFA), cholesterol, triglycerides, and phospholipids (Figure 2). Overall, both mouse models develop a metabolic phenotype consistent with T2D, with RCS10 mice exhibiting more pronounced weight gain and hyperinsulinemia and TH mice demonstrating broader dyslipidemia.

### 3.2 RCS10 And TH Mouse Models Develop DPN

**FIGURE 3:**
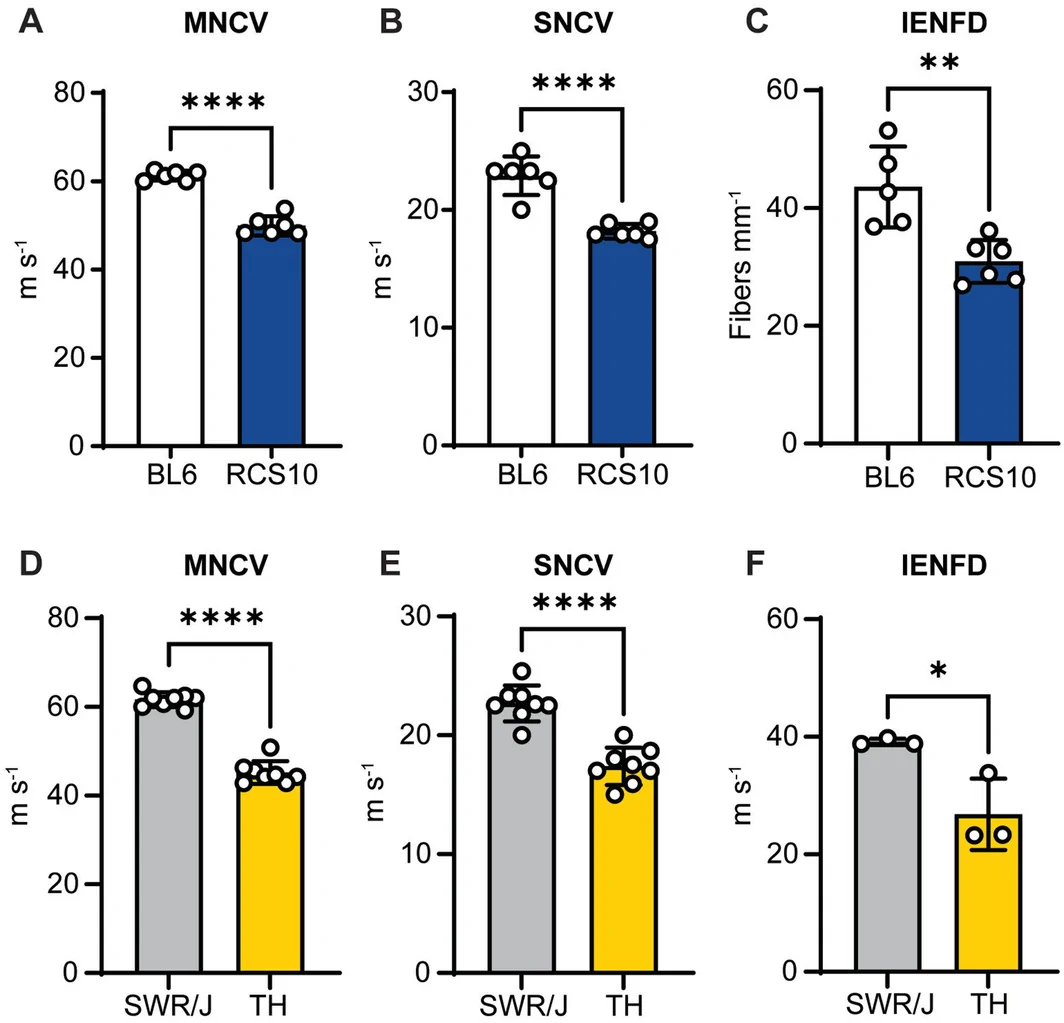
RCS10 and TH mice develop DPN. Terminal (A, D) motor nerve conduction velocities (MNCV), (B, E) sensory nerve conduction velocities (SNCV), and (C, F) intraepidermal nerve fiber densities (IENFD) in BL6 and RCS10 mice (A, B, *n* = 6; C, BL6, *n* = 5, RCS10, *n* = 6) and SWR/J and TH mice (D, E; *n* = 8; F, *n* = 3). Each data point represents an individual animal. Data are represented as mean ± standard deviation. Two‐tailed unpaired *t*‐test. *p < 0.05, **p < 0.01, ***p < 0.001, ****p < 0.0001.

We next assessed whether RCS10 and TH mice develop DPN at 24 weeks of age. Both models showed significant deficits in motor and sensory NCVs, indicating large fiber dysfunction compared to their respective controls (Figure 3A,B). Additionally, IENFDs were markedly reduced in both models (RCS10 vs. BL6, *p* = 0.004; TH vs. SWR/J, *p* = 0.025; Figure 3C), reflecting loss of distal small fiber innervation. Collectively, these findings demonstrate that both RCS10 and TH mice develop characteristic features of DPN [28].

### 3.3 Female TH Mice Do Not Develop DPN

Sex‐dependent differences in DPN have been reported, highlighting the need for experimental models that enable investigation of the mechanisms underlying these differences. Female RCS10 and TH mice develop obesity and hyperinsulinemia but generally do not progress to the hyperglycemia or glucose intolerance observed in males [29, 30]. Additionally, diabetic complications have not been well characterized in female mice from these models. To investigate potential sex‐dependent differences, we characterized the metabolic and neuropathic phenotypes of female TH mice. By 16 weeks of age, corresponding to the initial onset of the T2D phenotype in male TH mice [19] female TH mice had significantly greater body weight than female SWR/J controls (Figure S2A), whereas FBG levels did not differ between the groups (Figure S2B). We next assessed whether female TH mice developed DPN. Female TH mice had normal motor NCV and IENFD (Figure S2C,E) although sensory NCV was modestly reduced when compared to female SWR/J controls (*p* = 0.029; Figure S2D). Collectively, these findings demonstrate that female TH mice, unlike male TH mice, do not develop a robust DPN phenotype at this age.

### 3.4 Comparison of Polygenic RCS10 and TH Models to Established Prediabetic and T2D Models

To evaluate how the RCS10 and TH mice compare with established T2D models, we assessed disease severity metrics against published data from monogenic (*db/db*) and diet‐induced (HFD and HFD‐STZ) mouse models [20, 22]. For each metabolic and DPN parameter, we calculated the percent change relative to each model's respective control group (Table 1). TH mice did not gain as much weight as the monogenic and diet‐induced models, whereas RCS10 mice displayed significantly more pronounced hyperinsulinemia (RCS10 vs. TH, *p* = 0.005; RCS10 vs. *db/db*, *p* = 0.043, RCS10 vs. HFD‐STZ, *p* = 0.005; Table 1). Overall, the phenotypes of RCS10 and TH mice most closely resembled those of the HFD and HFD‐STZ models, while differing most from the *db/db* model, which showed greater changes in FBG, HbA1c, and both motor and sensory NCVs compared to its respective control (Table 1). As the *db/db* model is often criticized for exhibiting an excessively severe phenotype, our results suggest that the RCS10 and TH models may provide more moderate and clinically relevant alternatives [5].

**TABLE 1:** Comparison of polygenic, monogenic, and diet‐induced T2D mouse models.

Outcome	Model	Change from control (%)^a^ (95% CI)	*p* ^b^ Model versus RCS10	*p* ^b^ Model versus TH
BW (g)	RCS10	36.7 (15.0, 67.0)	**—**	**—**
	TH	17.8 (3.7, 35.9)	0.454	**—**
	*db/db* ^c^	52.6 (31.6, 80.0)	0.741	**0.040**
	HFD	60.1 (36.2, 92.0)	0.508	**0.017**
	HFD‐STZ	58.4 (34.4, 90.5)	0.569	**0.024**
HbA1c (%)	RCS10	19.8 (−8.9, 71.4)	**—**	**—**
	TH	42.9 (4.2, 115.6)	0.846	**—**
	*db/db*	235.0 (122.4, 453.7)	**0.002**	**0.014**
	HFD	2.1 (−16.4, 30.8)	0.779	0.221
	HFD‐STZ	6.3 (−14.5, 39.7)	0.913	0.338
Insulin (ng mL^−1^)	RCS10	9155.3 (314.9, 11038736.4)	**—**	**—**
	TH	−22.3 (−49.0, 102.4)	**0.005**	**—**
	*db/db*	54.9 (−21.4, 460.7)	**0.043**	0.423
	HFD	2051.2 (169.6, 149633.6)	0.933	**0.009**
	HFD‐STZ	−11.1 (−42.8, 115.3)	**0.005**	0.973
MNCV (m s^−1^)	RCS10	−17.0 (−20.0, −13.7)	**—**	**—**
	TH	−23.5 (−25.7, −21.1)	**0.004**	**—**
	*db/db*	−27.9 (−29.6, −26.0)	**< 0.001**	**0.010**
	HFD	−18.3 (−20.6, −15.9)	0.868	**0.008**
	HFD‐STZ	−18.2 (−20.6, −15.7)	0.899	**0.008**
SNCV (m s^−1^)	RCS10	−18.5 (−22.8, −13.6)	**—**	**—**
	TH	−20.9 (−24.4, −17.0)	0.795	**—**
	*db/db*	−27.8 (−30.4, −25.0)	**0.002**	**0.009**
	HFD	−21.5 (−24.6, −18.0)	0.624	0.989
	HFD‐STZ	−20.5 (−23.8, −16.8)	0.869	0.996
IENFDs (Fibers mm^−1^)	RCS10	−25.0 (−32.4, −15.2)	**—**	**—**
	TH	−19.9 (−29.8, −6.1)	0.866	**—**
	*db/db*	−24.6 (−32.9, −13.5)	> 0.999	0.814
	HFD	−19.4 (−26.6, −10.3)	0.737	> 0.999
	HFD‐STZ	−24.4 (−30.6, −16.6)	> 0.999	0.787

Abbreviations: BW, body weight; CI, confidence interval; HbA1c, hemoglobin A1c; HFD, high‐fat diet; HFD‐STZ, high‐fat diet with streptozotocin; IENFD, intraepidermal nerve fiber density; MNCV, motor nerve conduction velocity; SNCV, sensory nerve conduction velocity.

^a^ For each metabolic and DPN parameter, the disease model was compared to its corresponding control to calculate percent change.

^b^ ANOVA with linear contrast, comparison of model versus the corresponding RCS10 or TH outcomes, text in bold indicates *p* < 0.05.

^c^ Metabolic and neuropathy phenotyping data were extracted from previous studies of *db/db* (Eid et al., *FASEB J*, 2023), HFD, and HFD‐STZ mice (O'Brien et al., *Dis Model Mech*, 2018).

### 3.5 Metabolic Factors Correlate With DPN Parameters

To assess how metabolic health relates to DPN severity across models, we performed correlation analyses between metabolic and DPN parameters in each T2D model (Figure S3). FBG was negatively correlated with sensory NCVs in prediabetic and T2D mouse models and similarly showed a negative correlation with motor NCVs in all models except HFD. Body weight was also negatively correlated with both motor and sensory NCVs in all models except TH. Notably, while triglyceride levels were negatively correlated with motor and sensory NCVs and IENFDs in RCS10 and TH mice, they were positively associated with these measures in HFD and HFD‐STZ models. Overall, these findings indicate that metabolic factors are broadly linked to DPN severity across T2D models. Importantly, the correlation patterns observed in RCS10 mice align with those reported in human T2D DPN, supporting its value for translational studies.

### 3.6 Caloric Restriction Supports Metabolic Health in the RCS10 Mouse Model of DPN

**FIGURE 4:**
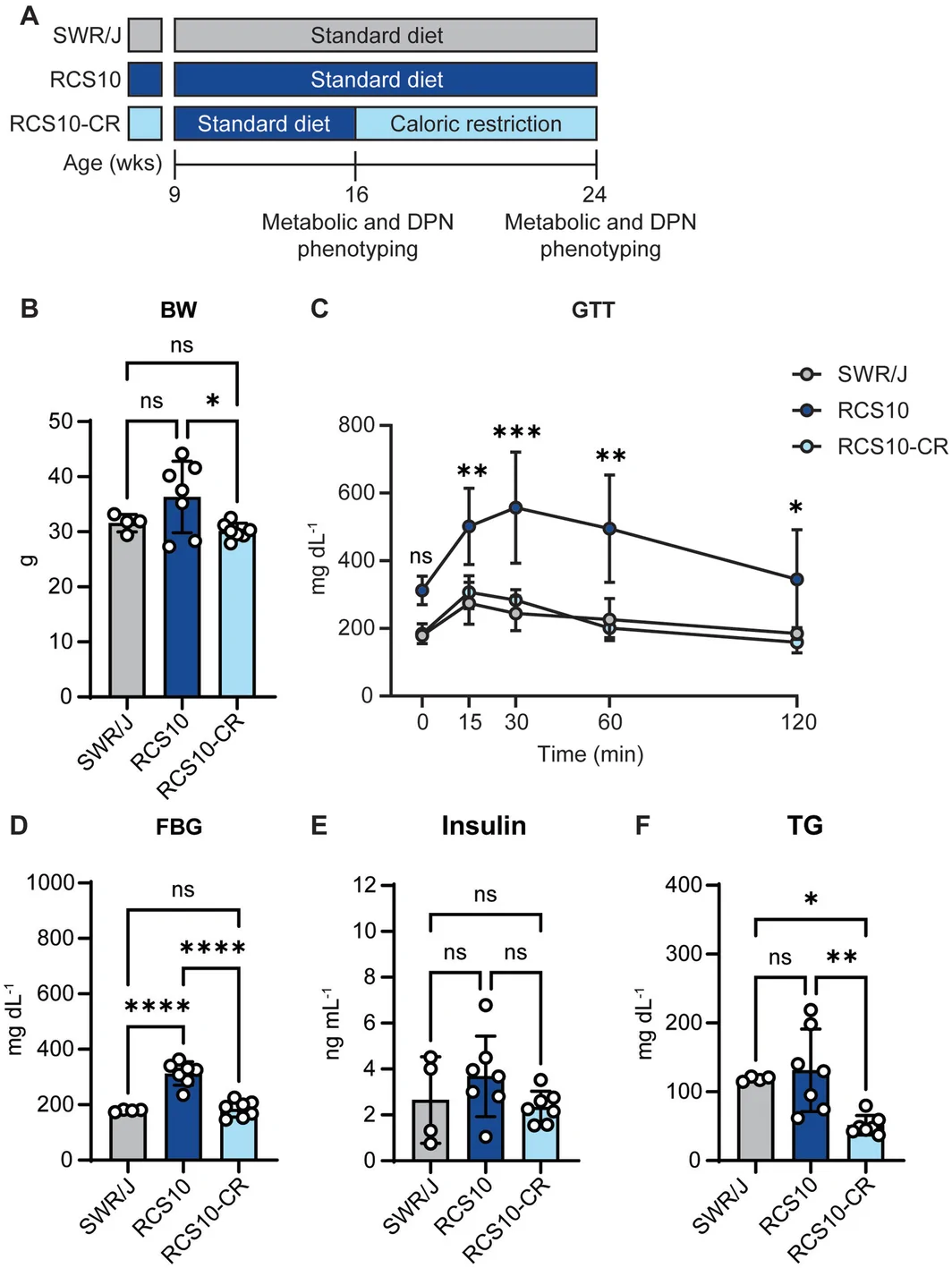
Dietary restriction improves metabolic dysfunction in RCS10 mice. (A) Study design for dietary intervention paradigm. Nine‐week‐old RCS10 and control SWR/J mice were fed a standard diet. At 16 weeks of age, half of the RCS10 mice were switched to a 60% calorie restricted diet. Body weight (BW) and fasting blood glucose (FBG) were recorded biweekly. Baseline glucose tolerance testing (GTT) and diabetic peripheral neuropathy (DPN) phenotyping (nerve conduction velocities [NCVs]) were measured at 16 weeks of age. Terminal GTT, HbA1C, plasma (insulin and lipids), body composition, and DPN phenotyping (NCVs and intraepidermal nerve fiber densities) were assessed at 24 weeks of age. Terminal (B) BW, (C) GTT, (D) FBG, (E) insulin, and (F) triglycerides (TG) in SWR/J (*n* = 4), RCS10 (*n* = 7), and RCS10‐CR (*n* = 7) mice. Each data point represents an individual animal. Data are represented as mean ± standard deviation. Data in (B, D–F) assessed by one‐way ANOVA with Tukey's multiple comparisons and (C) by repeated measures two‐way ANOVA with Tukey's multiple comparisons. For (C), * indicates a significant difference compared to SWR/J. ns, not significant, *p < 0.05, **p < 0.01, ***p < 0.001, ****p < 0.0001.

In the second part of our study, we evaluated the efficacy of a dietary intervention in improving metabolic health and DPN in a polygenic mouse model of DPN. Although both RCS10 and TH strains displayed comparable metabolic and DPN profiles, we selected RCS10 for subsequent experiments due to ease of breeding, greater availability, and lower activity, which facilitates phenotyping. Additionally, the correlation patterns observed in RCS10 most closely resemble those reported in human T2D DPN, supporting its translational relevance. In this interventional paradigm, RCS10 mice were monitored from 9 to 16 weeks of age, during which they developed both metabolic dysfunction and features of DPN (Figure 4A; Figure S4A,D,E and S5). At 16 weeks, mice were either placed on a calorie‐restricted diet (60% of standard diet intake) or continued on an *ad libitum* diet. Age‐matched SWR/J mice maintained on an *ad libitum* diet served as healthy controls.

Caloric restriction (CR) reduced longitudinal and terminal body weight (*p* = 0.04; Figure 4B; Figure S4A) with RCS10‐CR mice exhibiting lower fat mass and increased lean mass compared to RCS10 animals at study termination (Figure S4B,C). Terminal glucose tolerance and FBG levels in RCS10‐CR mice were like those of SWR/J controls (Figure 4C,D), while terminal plasma insulin levels did not differ significantly across groups (Figure 4E). Moreover, caloric restriction improved the lipid profile, with reduced triglyceride and cholesterol levels compared to SWR/J and RCS10 mice (Figure 4F; Figure S6). Taken together, these findings suggest that caloric restriction favorably impacts metabolic health in the RCS10 mouse model of DPN.

### 3.7 Caloric Restriction Restores Large Fiber Function in the RCS10 Mouse Model of DPN

**FIGURE 5:**
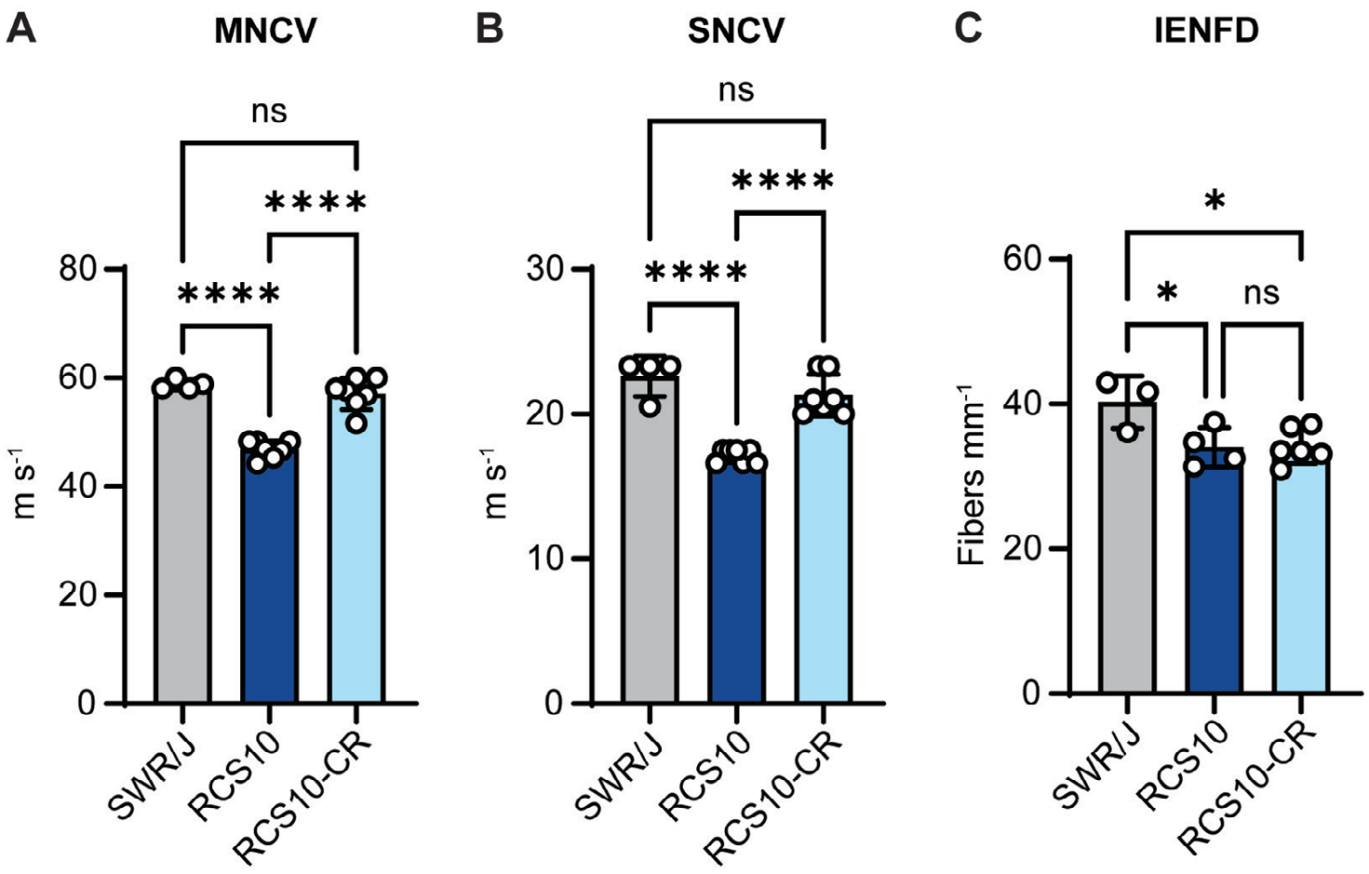
Caloric restriction restores large fiber function in RCS10 mice. Terminal (A) motor nerve conduction velocities (MNCV), (B) sensory nerve conduction velocities (SNCV), and (C) intraepidermal nerve fiber densities (IENFD) in SWR/J (A, B, *n* = 4; C, *n* = 3), RCS10 (A, B, *n* = 7; C, *n* = 4), and RCS10‐CR (A, B, *n* = 7; C, *n* = 6) mice. Each data point represents an individual animal. Data are represented as mean ± standard deviation. One‐way ANOVA with Tukey's multiple comparisons. ns, not significant, *p < 0.05, ****p < 0.0001.

By the end of the study, caloric restriction restored large fiber function, as evidenced by normalized motor and sensory NCVs (Figure 5A,B). However, terminal IENFDs remained unchanged between RCS10 and RCS10‐CR animals, indicating no improvement in small fiber neuropathy (*p* = 0.995; Figure 5C). These findings suggest that while caloric restriction can restore certain aspects of DPN, it cannot ameliorate all features of the disease.

## 4 Discussion

Although existing animal models of DPN have advanced our understanding of disease pathophysiology, they have not yielded effective therapies, underscoring the need for models that better recapitulate human DPN and support translational discovery. In this study, we characterized the metabolic and neuropathic phenotypes of two polygenic T2D models, RCS10 and TH. We further applied a caloric restriction intervention in the RCS10 model to determine whether improving metabolic health could ameliorate neuropathic deficits.

Clinically, patients progress gradually from prediabetes to overt T2D [3]. In contrast, monogenic mouse models rapidly become hyperglycemic, often within 4 weeks, and lack an extended prediabetic phase [5, 31]. Diet‐induced models more closely mirror the slow metabolic deterioration observed in humans [5] but exhibit substantial variability due to strain background, dietary fat content, and fat source, which can limit reproducibility across studies [5, 6, 7, 8, 9, 10, 11, 12, 13, 14]. Like diet‐induced models, RCS10 and TH mice showed a gradual onset of metabolic dysfunction and delayed hyperglycemia, with milder changes in HbA1c and FBG compared to *db/db* mice. Notably, diabetes developed without severe obesity, consistent with many individuals with T2D [32]. Dyslipidemia, a major contributor to DPN risk [3, 4, 5, 6, 7, 8, 9, 10, 11, 12, 13, 14, 15, 16, 17, 18, 19, 20, 21, 22, 23, 24, 25, 26, 27, 28, 29, 30, 31, 32, 33] is not consistently present in commonly used monogenic or diet‐induced models [6, 7, 8, 9, 10, 11, 12, 13, 14, 15, 16, 17, 18, 19, 20, 21, 22, 23, 24, 25, 26, 27, 28, 29, 30, 31, 32, 33, 34, 35, 36]. In contrast, both RCS10 and TH mice developed increased triglycerides, more accurately reflecting lipid disturbances observed in human T2D [37, 38, 39].

While RCS10 and TH mice are established models of T2D, their neuropathic features were previously uncharacterized. We demonstrate that both strains develop hallmark features of DPN, including large fiber impairment and small fiber loss, meeting consensus criteria outlined in [28]. Compared with other models, *db/db* mice displayed the most severe large fiber dysfunction, whereas RCS10 and TH neuropathy resembled the more moderate phenotype of diet‐induced models. Monogenic models develop rapid, early, and often intervention‐resistant neuropathy [7, 8, 9, 10, 11, 12, 13, 14, 15, 16, 17, 18, 19, 20, 21, 22, 23, 24, 25, 26, 27, 28, 29, 30, 31, 32, 33, 34, 35, 36, 37, 38, 39, 40, 41], while diet‐induced models vary in neuropathy onset and severity [5]. In contrast, RCS10 and TH mice develop DPN independent of dietary triggers, providing a genetically driven platform in which metabolic and neuropathic decline progress gradually, features that more closely parallel human DPN.

To further evaluate their clinical relevance, we examined associations between metabolic factors and DPN outcomes. FBG was inversely correlated with motor and sensory NCVs, and body weight showed similar inverse relationships in all strains except TH. These patterns align with clinical evidence that elevated FBG and higher body mass index (BMI) are associated with reduced NCVs and increased DPN risk [42, 43, 44, 45], and with reports identifying glycemic fluctuations and central obesity as recognized predictors of DPN in individuals with T2D [46, 47, 48]. Collectively, these data suggest that RCS10 and TH mice recapitulate key metabolic‐neuropathic relationships observed in human DPN. Triglycerides were negatively correlated with NCVs in RCS10 and TH mice, mirroring clinical observations linking hypertriglyceridemia to DPN severity and progression [37, 49], whereas diet‐induced models showed opposite associations. Despite species‐specific differences in lipid metabolism [50], RCS10 and TH mice appear to better capture the pathophysiological link between dyslipidemia and DPN than conventional diet‐induced models.

We next assessed whether caloric restriction could improve metabolic and neuropathic outcomes in RCS10 mice. Consistent with previous findings in monogenic and diet‐induced models [21, 22], caloric restriction reduced body weight, improved glucose tolerance, lowered FBG, and decreased triglyceride levels. These metabolic improvements were accompanied by restored large fiber function, as reflected by normalized NCVs, whereas IENFDs remained unchanged. Although several preclinical and clinical studies have shown that small fiber innervation can improve with metabolic or lifestyle interventions, particularly in prediabetic or early diabetic settings [51, 52], structural recovery appears to depend on disease stage and the duration of metabolic improvement. Clinical studies have demonstrated persistent IENFD loss despite sustained metabolic normalization in individuals with established DPN [53, 54], whereas earlier intervention has been associated with measurable small‐fiber regeneration [55]. Similarly, dietary weight loss in individuals with severe obesity improved neuropathy symptoms while stabilizing, rather than restoring, IENFD [56]. Additionally, prior work in *db/db* mice [22] has similarly failed to demonstrate IENFD recovery despite concurrent metabolic benefits. Thus, the absence of small fiber recovery in RCS10 mice may reflect the stage of neuropathy at intervention onset, the relatively short intervention duration, and/or model‐specific differences in regenerative capacity. Overall, these findings indicate that the RCS10 model is responsive to dietary modulation and that caloric restriction can enhance metabolic control and large fiber nerve function, supporting the utility of this model for evaluating therapeutic strategies for DPN.

Our study has limitations. The primary studies were conducted in male mice, although sex differences have been reported in metabolic and neuropathic phenotypes, including the onset of diabetes, insulin resistance, nerve fiber injury, and development of pain [5, 57, 58]. This sex‐specific design for both monogenic strains was based primarily on the observation that male RCS and TH mice, unlike their female counterparts, develop a diabetic phenotype. Consistent with this sexually dimorphic phenotype, female TH mice did not develop neuropathy, emphasizing the importance of incorporating sex as a biological variable in DPN studies. IENFDs for TH mice were measured in a separate cohort; while statistically matching preserved treatment effects, individual‐level biological variation could not be assessed. Behavioral assessments of mechanical and thermal sensitivity could not be performed as the animals were markedly hyperactive, which precluded the reliable assessment of sensory behaviors. Our study, however, remains consistent with Neurodiab consensus recommendations by incorporating complementary electrophysiological and structural endpoints to confirm neuropathy [28]. Finally, caloric restriction was not tested in TH mice; future studies are needed to evaluate intervention responsiveness in this strain.

Mouse models remain essential for elucidating DPN mechanisms and for therapeutic development, yet many fail to capture the complexity and gradual progression of human disease. Our findings identify the polygenic male RCS10 and TH strains as promising alternatives that more closely mirror the metabolic deterioration and progressive neuropathy characteristic of human DPN. We propose that these models, particularly the diet‐responsive RCS10 strain, provide robust platforms for investigating disease mechanisms and evaluating candidate therapeutic strategies aimed at preventing or slowing nerve injury in DPN.

## Author Contributions

Stéphanie A. Eid, John M. Hayes, Phillipe D. O'Brien, and Eva L. Feldman conceived and designed the research. Stéphanie A. Eid, Andrew D. Carter, John M. Hayes, Diana M. Rigan, Chloe Kiriluk, Crystal M. Pacut, and Phillipe D. O'Brien performed the research and acquired the data. Stéphanie A. Eid, Andrew D. Carter, Emily J. Koubek, Jihyun Park, and Dae‐Gyu Jang analyzed and interpreted the data. Stéphanie A. Eid and Emily J. Koubek wrote the original draft of the manuscript. All authors were involved in revising the manuscript.

## Funding

This work was supported by HHS | National Institutes of Health (NIH), P30DK020572, K01DK135799, R01DK130913, R24DK082841. Novo Nordisk Foundation, NNF14OC0011633. Nathan and Rose Milstein Research Fund, N/A. Sinai Medical Staff Foundation (SMSF), N/A. Robert and Katherine Jacobs Environmental Health Initiative, N/A. Andrea and Lawrence Wolfe Brain Health Initiative, N/A. Dr. John H. Doran Neuropathy Research Fund, N/A. NeuroNetwork for Emerging Therapies at the University of Michigan, N/A. Medical Practice Plan Grant from the American University of Beirut, N/A.

## Conflicts of Interest

The authors declare no conflicts of interest.

## Supporting information


**Figure S1:** Longitudinal body weight and fasting blood glucose in RCS10 and TH mice. Longitudinal (A–C) body weight (BW) and (B–D) fasting blood glucose (FBG) in RCS10 and BL6 mice (A, B; *n* = 6) and TH and SWR/J mice (C, D; *n* = 6). Each data point represents an individual animal. Data are represented as mean ± standard deviation. Repeated measures two‐way ANOVA with Tukey's multiple comparison. ns, not significant, *p < 0.05, **p < 0.01, ***p < 0.001.
**Figure S2:** Metabolic and neuropathic phenotyping of female TH mice. Terminal (A) body weight (BW), (B) fasting blood glucose (FBG), (C) motor nerve conduction velocities (MNCV), (D) sensory nerve conduction velocities (SNCV), and (E) intraepidermal nerve fiber densities (IENFD) were measured in female TH (*n* = 6) and SWR/J mice (*n* = 6) at 16 weeks of age. Each data point represents an individual animal. Data are presented as mean ± standard deviation. Two‐tailed unpaired t‐test. ns, not significant, *p < 0.05, ****p < 0.0001.
**Figure S3:** Metabolic factors correlate to DPN parameters in polygenic, monogenic, and diet‐induced T2D mouse models. Pearson's correlation coefficient analysis of metabolic factors to DPN parameters in RCS10 (polygenic; A–G, *n* = 6), TH (polygenic; A, B, *n* = 9; C, D, *n* = 6; E, F, *n* = 8; G, *n* = 3), db/db (monogenic; A–F, *n* = 10; G, *n* = 4), high‐fat diet (HFD; diet‐induced; A–C, E, F, *n* = 10; D, *n* = 8; G, *n* = 9) and HFD with streptozotocin (HFD‐STZ; diet‐induced; A, B, D–G, *n* = 9; C, *n* = 8) T2D mouse models. A statistical matching method was employed for data from TH mice. Color scale represents positive (red) and negative (blue) correlations. * indicates significance. *p < 0.05, **p < 0.01, ***p < 0.001. BW, body weight; CHOL, cholesterol; Corr, correlation; FBG, fasting blood glucose; IENFD, intraepidermal nerve fiber density; MNCV, motor nerve conduction velocity; NEFA, non‐esterified fatty acids; SNCV, sensory nerve conduction velocity; TG, triglycerides; PPL, phospholipids.
**Figure S4:** Metabolic phenotyping in dietary intervention paradigm. (A) Longitudinal body weight (BW) in all study groups (SWR/J, *n* = 4; RCS10 and RCS10‐CR, *n* = 7). Dotted line indicates initiation of dietary intervention. Repeated measures two‐way ANOVA with Tukey's multiple comparisons. * indicates a significant difference between SWR/J and RCS10. *p < 0.05, **p < 0.01, ***p < 0.001. * indicates a significant difference between SWR/J and RCS10‐CR. ***p < 0.001, ****p < 0.0001. (B, C) Terminal fat (B) and lean (C) mass at study end (SWR/J, *n* = 4; RCS10 and RCS10‐CR, *n* = 7). One‐way ANOVA with Tukey's multiple comparisons. *p < 0.05, **p < 0.01. (D) Baseline glucose tolerance testing (GTT) at 16 weeks of age, prior to initiation of dietary intervention (SWR/J, *n* = 4; RCS10, *n* = 14). Repeated measures two‐way ANOVA with Sidak's multiple comparisons. ***p < 0.001, ****p < 0.0001. (E) Longitudinal fasting blood glucose in all study groups (SWR/J, *n* = 4; RCS10 and RCS10‐CR, *n* = 7). Dotted line indicates initiation of dietary intervention. Repeated measures two‐way ANOVA with Tukey's multiple comparison. * indicates a significant difference between SWR/J and RCS10. *p < 0.05, **p < 0.01, ***p < 0.001. * indicates a significant difference between SWR/J and RCS10‐CR. ***p < 0.001. Each data point represents an individual animal. Data are represented as mean ± s.d. ns, not significant.
**Figure S5:** Nerve conduction velocities prior to caloric restriction in RCS10 mice. Baseline (A) motor nerve conduction velocities (MNCV) and (B) sensory nerve conduction velocities (SNCV) at 16 weeks of age, prior to initiation of dietary interventions. (SWR/J, *n* = 4; RCS10, *n* = 14). Each data point represents an individual animal. Data are represented as mean ± s.d. Two‐tailed unpaired t‐test. **p < 0.01, ***p < 0.001.
**Figure S6:** Lipid profiles following caloric restriction in RCS10 mice. Terminal (A) non‐esterified fatty acid (NEFA), (B) cholesterol (CHOL), and (C) phospholipids in SWR/J (*n* = 4), RCS10 (*n* = 7), and RCS10‐CR (*n* = 7) mice. Each data point represents an individual animal. Data are represented as mean ± s.d. One‐way ANOVA with Tukey's multiple comparisons. ns, not significant, *p < 0.05.

**Table S1:** Statistical assumption checks for analyses presented in Figures 1, 2, 3, 4, 5 and Figures S1, S2, and Figures S4–S6.
**Table S2:** Sensitivity analysis for data presented in Figures 1–3 and Figures S2–S5.
**Table S3:** Sensitivity analysis for data presented in Figures 4 and 5 and Figures S4–S6.
**Table S4:** Sensitivity analysis for data presented in Figure 4 and Figures S1–S4.

## Data Availability

Included in article.
