# Supplementary material for: NONcNZO10/LtJ and TALLY‐HO/JngJ Mice as Novel Models of Diabetic Peripheral Neuropathy
Source: FASEB J. 2026 Sep 5;40(17):e72275. doi: 10.1096/fj.202600848R (PMC13545661; doi:10.1096/fj.202600848R)
Supplement: Supplementary file 1 [file FSB2-40-e72275-s001.docx]

**Fig. S1. Longitudinal body weight and fasting blood glucose in RCS10 and TH mice.** Longitudinal (**A**, **C**) body weight (BW) and (**B**, **D**) fasting blood glucose (FBG) in RCS10 and BL6 mice (**A**, **B**; n=6) and TH and SWR/J mice (**C**, **D**; n=6). Each data point represents an individual animal. Data are represented as mean±s.d.. Repeated measures two-way ANOVA with Tukey’s multiple comparison. ns, not significant, **p*<0.05, ***p*<0.01, ****p*<0.001.

**Fig S2. Metabolic and neuropathic phenotyping of female TH mice.** Terminal (**A**) body weight (BW), (**B**) fasting blood glucose (FBG), (**C**) motor nerve conduction velocities (MNCV), (**D**) sensory nerve conduction velocities (SNCV), and (**E**) intraepidermal nerve fiber densities (IENFD) were measured in female TH (n=6) and SWR/J mice (n=6) at 16 weeks of age. Each data point represents an individual animal. Data are presented as mean±s.d. Two-tailed unpaired t-test. ns, not significant, **p*<0.05, *****p*<0.0001.

**Fig. S3. Metabolic factors correlate to DPN parameters in polygenic, monogenic, and diet-induced T2D mouse models.** Pearson’s correlation coefficient analysis of metabolic factors to DPN parameters in RCS10 (polygenic; **A**-**G,** n=6), TH (polygenic; **A**, **B**, n=9; **C**, **D**, n=6; **E**, **F**, n=8; **G**, n=3), *db/db* (monogenic; **A**-**F**, n=10; **G**, n=4), high-fat diet (HFD; diet-induced; **A**-**C**, **E**, **F**, n=10; **D**, n=8; **G**, n=9) and HFD with streptozotocin (HFD-STZ; diet-induced; **A**, **B**, **D**-**G**, n=9; **C**, n=8) T2D mouse models. A statistical matching method was employed for data from TH mice. Color scale represents positive (red) and negative (blue) correlations. * indicates significance. **p*<0.05, ***p*<0.01, ****p*<0.001. BW, body weight; CHOL, cholesterol; Corr, correlation; FBG, fasting blood glucose; IENFD, intraepidermal nerve fiber density; MNCV, motor nerve conduction velocity; NEFA, non-esterified fatty acids; SNCV, sensory nerve conduction velocity; TG, triglycerides; PPL, phospholipids.

**Fig. S4. Metabolic phenotyping in dietary intervention paradigm.** (**A**) Longitudinal body weight (BW) in all study groups (SWR/J, n=4; RCS10 and RCS10-CR, n=7). Dotted line indicates initiation of dietary intervention. Repeated measures two-way ANOVA with Tukey’s multiple comparisons. * indicates a significant difference between SWR/J and RCS10. **p*<0.05, ***p*<0.01, ****p*<0.001. ^#^ indicates a significant difference between SWR/J and RCS10-CR. ^###^*p*<0.001, ^####^*p*<0.0001. (**B**, **C**) Terminal fat (**B**) and lean (**C**) mass at study end (SWR/J, n=4; RCS10 and RCS10-CR, n=7). One-way ANOVA with Tukey’s multiple comparisons. **p*<0.05, ***p*<0.01. (**D**) Baseline glucose tolerance testing (GTT) at 16 weeks of age, prior to initiation of dietary intervention (SWR/J, n=4; RCS10, n=14). Repeated measures two-way ANOVA with Sidak’s multiple comparisons. ****p*<0.001, *****p*<0.0001. (**E**) Longitudinal fasting blood glucose in all study groups (SWR/J, n=4; RCS10 and RCS10-CR, n=7). Dotted line indicates initiation of dietary intervention. Repeated measures two-way ANOVA with Tukey’s multiple comparison. * indicates a significant difference between SWR/J and RCS10. **p*<0.05, ***p*<0.01, ****p*<0.001. ^#^ indicates a significant difference between SWR/J and RCS10-CR. ^###^*p*<0.001. Each data point represents an individual animal. Data are represented as mean±s.d.. ns, not significant.

**Fig. S5. Nerve conduction velocities prior to caloric restriction in RCS10 mice.** Baseline (**A**) motor nerve conduction velocities (MNCV) and (**B**) sensory nerve conduction velocities (SNCV) at 16 weeks of age, prior to initiation of dietary interventions. (SWR/J, n=4; RCS10, n=14). Each data point represents an individual animal. Data are represented as mean±s.d.. Two-tailed unpaired t-test. ***p*<0.01, ****p*<0.001.

**Fig. S6. Lipid profiles following caloric restriction in RCS10 mice.** Terminal (**A**) non-esterified fatty acid (NEFA), (**B**) cholesterol (CHOL), and (**C**) phospholipids in SWR/J (n=4), RCS10 (n=7), and RCS10-CR (n=7) mice. Each data point represents an individual animal. Data are represented as mean±s.d.. One-way ANOVA with Tukey’s multiple comparisons. ns, not significant, **p*<0.05.
