# Supplementary material for: NONcNZO10/LtJ and TALLY‐HO/JngJ Mice as Novel Models of Diabetic Peripheral Neuropathy
Source: FASEB J. 2026 Sep 5;40(17):e72275. doi: 10.1096/fj.202600848R (PMC13545661; doi:10.1096/fj.202600848R)
Supplement: Supplementary file 2 [file FSB2-40-e72275-s002.docx]

**Table S1. Statistical assumption checks for analyses presented in Figures 1-5 and Supplemental Figures 1, 2, and 4-6**

| **BL6 vs. RCS10** | | | | | |
| --- | --- | --- | --- | --- | --- |
| Variable | Levene or LRT *p*-value | Equal variance | Shapiro *p*-value | Normality |  |
| BW^†^ | 0.394 | Satisfied | 0.143 | Satisfied |  |
| FBG | 0.331 | Satisfied | 0.165 | Satisfied |  |
| HbA1c | 0.142 | Satisfied | 0.635 | Satisfied |  |
| Insulin | 0.095 | Satisfied | **0.008**^‡^ | Violated |  |
| NEFA | 0.882 | Satisfied | 0.898 | Satisfied |  |
| CHOL | 0.909 | Satisfied | 0.288 | Satisfied |  |
| TG | 0.980 | Satisfied | 0.226 | Satisfied |  |
| PPL | 0.349 | Satisfied | 0.130 | Satisfied |  |
| MNCV | 0.274 | Satisfied | 0.075 | Satisfied |  |
| SNCV | 0.366 | Satisfied | 0.109 | Satisfied |  |
| IENFD | 0.219 | Satisfied | 0.823 | Satisfied |  |
| Longitudinal BW | **0.003** | Violated | 0.735 | Satisfied |  |
| Longitudinal FBG | **<0.001** | Violated | 0.199 | Satisfied |  |
| **SWR/J vs. TH** | | | | | |
| Variable | Levene or LRT *p*-value | Equal variance | Shapiro *p*-value | Normality |  |
| BW | 0.211 | Satisfied | **0.048** | Violated |  |
| FBG | **0.049** | Violated | **0.001** | Violated |  |
| HbA1c | **0.017** | Violated | 0.105 | Satisfied |  |
| Insulin | 0.363 | Satisfied | 0.075 | Satisfied |  |
| NEFA | 0.119 | Satisfied | 0.882 | Satisfied |  |
| CHOL | **0.022** | Violated | 0.887 | Satisfied |  |
| TG | **0.035** | Violated | 0.709 | Satisfied |  |
| PPL | 0.425 | Satisfied | 0.407 | Satisfied |  |
| MNCV | 0.536 | Satisfied | **0.046** | Violated |  |
| SNCV | 0.724 | Satisfied | 0.548 | Satisfied |  |
| IENFD | 0.414 | Satisfied | 0.154 | Satisfied |  |
| Longitudinal BW | **<0.001** | Violated | **0.005** | Violated |  |
| Longitudinal FBG | **<0.001** | Violated | **<0.001** | Violated |  |
| **Female SWR/J vs. Female TH** | | | | |  |
| Variable | Levene *p*-value | Equal variance | Shapiro *p*-value | Normality |  |
| BW | 0.810 | Satisfied | 0.578 | Satisfied |  |
| FBG | 0.853 | Satisfied | 0.127 | Satisfied |  |
| MNCV | 0.369 | Satisfied | **0.002** | Violated |  |
| SNCV | **0.043** | Violated | 0.080 | Satisfied |  |
| IENFD | 0.873 | Satisfied | 0.983 | Satisfied |  |
| **SWR/J vs. RCS10 vs. RCS10-CR** | | | | | |
| Variable | Levene or LRT *p*-value | Equal variance | Shapiro *p*-value | Normality |  |
| BW | **0.017** | Violated | 0.148 | Satisfied |  |
| Longitudinal GTT | **<0.001** | Violated | **0.012** | Violated |  |
| FBG | 0.185 | Satisfied | 0.638 | Satisfied |  |
| Insulin | **0.022** | Violated | 0.854 | Satisfied |  |
| TG | **0.007** | Violated | **0.041** | Violated |  |
| MNCV | 0.335 | Satisfied | 0.188 | Satisfied |  |
| SNCV | 0.466 | Satisfied | 0.515 | Satisfied |  |
| IENFD | 0.896 | Satisfied | 0.451 | Satisfied |  |
| Longitudinal BW | **<0.001** | Violated | **<0.001** | Violated |  |
| Fat mass | 0.076 | Satisfied | 0.759 | Satisfied |  |
| Lean mass | 0.055 | Satisfied | 0.446 | Satisfied |  |
| Baseline GTT | **<0.001** | Violated | **0.018** | Violated |  |
| Longitudinal FBG | **0.001** | Violated | 0.209 | Satisfied |  |
| Baseline MNCV | 0.291 | Satisfied | **0.001** | Violated |  |
| Baseline SNCV | 0.077 | Satisfied | **0.001** | Violated |  |
| NEFA | **0.029** | Violated | 0.580 | Satisfied |  |
| CHOL | 0.509 | Satisfied | 0.833 | Satisfied |  |
| PPL | 0.333 | Satisfied | 0.086 | Satisfied |  |

^†^ Abbreviations: BW, body weight; FBG, fasting blood glucose; HbA1c, hemoglobin A1c; NEFA, non-esterified fatty acid; CHOL, cholesterol; TG, triglyceride; PPL, phospholipid; MNCV, motor nerve conduction velocity; SNCV, sensory nerve conduction velocity; IENFD, intraepidermal nerve fiber density; GTT, glucose tolerance, test; LRT, likelihood ratio tests.

^‡^ Bold text indicates *p*<0.05.

**Table S2. Sensitivity analysis for data presented in Figures 1-3 and Supplemental Figures 2 and 5**

| **BL6 vs. RCS10** | | | |
| --- | --- | --- | --- |
| Variable | Student's T-test | Welch's T-test | Wilcoxon Rank-sum Test |
| BW^†^ | **<0.001**^‡^ | **<0.001** | **0.002** |
| FBG | **0.003** | **0.004** | **0.005** |
| HbA1c | **<0.001** | **0.001** | **0.004** |
| Insulin | **0.004** | **0.012** | **0.002** |
| NEFA | 0.202 | 0.202 | 0.310 |
| CHOL | 0.152 | 0.152 | 0.149 |
| TG | **<0.001** | **<0.001** | **0.002** |
| PPL | **0.012** | **0.012** | **0.016** |
| MNCV | **<0.001** | **<0.001** | **0.005** |
| SNCV | **<0.001** | **<0.001** | **0.004** |
| IENFD | **0.004** | **0.011** | **0.004** |
| **SWR/J vs. TH** | | | |
| Variable | Student's T-test | Welch's T-test | Wilcoxon Rank-sum Test |
| BW | **0.005** | **0.008** | **0.002** |
| FBG | **<0.001** | **<0.001** | **0.002** |
| HbA1c | **0.005** | **0.016** | **0.005** |
| Insulin | 0.858 | 0.860 | 0.378 |
| NEFA | **<0.001** | **<0.001** | **0.002** |
| CHOL | **0.002** | **0.004** | **0.008** |
| TG | **0.009** | **0.019** | **0.009** |
| PPL | **0.021** | **0.021** | **0.037** |
| MNCV | **<0.001** | **<0.001** | **<0.001** |
| SNCV | **<0.001** | **<0.001** | **0.001** |
| IENFD | **0.025** | 0.071 | 0.100 |
| **Female SWR/J vs. Female TH** | | | |
| Variable | Student's T-test | Welch's T-test | Wilcoxon Rank-sum Test |
| BW | **<0.001** | **<0.001** | **0.002** |
| FBG | 0.534 | 0.534 | 0.520 |
| MNCV | 0.559 | 0.568 | 0.064 |
| SNCV | **0.029** | **0.032** | 0.054 |
| IENFD | 0.543 | 0.543 | 0.937 |
| **SWR/J vs. RCS10 vs. RCS10-CR** | | | |
| Variable | Student's T-test | Welch's T-test | Wilcoxon Rank-sum Test |
| Baseline MNCV | **<0.001** | **<0.001** | **0.012** |
| Baseline SNCV | **0.001** | 0.091 | **0.006** |

^†^ Abbreviations: BW, body weight; FBG, fasting blood glucose; HbA1c, hemoglobin A1c; NEFA, non-esterified fatty acid; CHOL, cholesterol; TG, triglyceride; PPL, phospholipid; MNCV, motor nerve conduction velocity; SNCV, sensory nerve conduction velocity; IENFD, intraepidermal nerve fiber density.

^‡^ Bold text indicates *p*<0.05.

**Table S3. Sensitivity analysis for data presented in Figures 4 and 5 and Supplemental Figure 4 and 6**

| **SWR/J vs. RCS10 vs. RCS10-CR** | | | | | |
| --- | --- | --- | --- | --- | --- |
| Variable | Group 1 | Group 2 | One-way ANOVA with equal-variance^†^ | One-way ANOVA with unequal-variance^‡^ | Kruskal-Wallis Test^§^ |
| BW | RCS10-CR | RCS10 | **0.040** | 0.100 | 0.215 |
|  | RCS10-CR | SWR/J | 0.850 | 0.351 | 0.775 |
|  | RCS10 | SWR/J | 0.211 | 0.224 | 0.775 |
| FBG | RCS10-CR | RCS10 | **<0.001** | **<0.001** | **0.007** |
|  | RCS10-CR | SWR/J | 0.956 | 0.868 | 0.814 |
|  | RCS10 | SWR/J | **<0.001** | **<0.001** | **0.009** |
| Insulin | RCS10-CR | RCS10 | 0.469 | 0.357 | 0.558 |
|  | RCS10-CR | SWR/J | 0.916 | 0.948 | >0.999 |
|  | RCS10 | SWR/J | 0.796 | 0.883 | >0.999 |
| TG | RCS10-CR | RCS10 | **0.004** | **0.028** | **0.007** |
|  | RCS10-CR | SWR/J | **0.037** | **<0.001** | **0.028** |
|  | RCS10 | SWR/J | 0.866 | 0.849 | 0.881 |
| MNCV | RCS10-CR | RCS10 | **<0.001** | **<0.001** | **0.008** |
|  | RCS10-CR | SWR/J | 0.466 | 0.405 | 0.478 |
|  | RCS10 | SWR/J | **<0.001** | **<0.001** | **0.005** |
| SNCV | RCS10-CR | RCS10 | **<0.001** | **<0.001** | **0.008** |
|  | RCS10-CR | SWR/J | 0.197 | 0.363 | 0.436 |
|  | RCS10 | SWR/J | **<0.001** | **0.006** | **0.004** |
| IENFD | RCS10-CR | RCS10 | 0.995 | 0.995 | 0.974 |
|  | RCS10-CR | SWR/J | **0.029** | 0.156 | 0.182 |
|  | RCS10 | SWR/J | **0.037** | 0.148 | 0.182 |
| Fat mass | RCS10-CR | RCS10 | **<0.001** | **0.002** | **0.004** |
|  | RCS10-CR | SWR/J | 0.911 | 0.928 | 0.710 |
|  | RCS10 | SWR/J | **0.003** | **0.042** | **0.035** |
| Lean mass | RCS10-CR | RCS10 | **0.023** | **0.035** | **0.043** |
|  | RCS10-CR | SWR/J | 0.719 | 0.284 | 0.506 |
|  | RCS10 | SWR/J | 0.146 | 0.147 | 0.506 |
| NEFA | RCS10-CR | RCS10 | 0.200 | 0.296 | 0.949 |
|  | RCS10-CR | SWR/J | 0.996 | 0.964 | 0.957 |
|  | RCS10 | SWR/J | 0.265 | 0.260 | 0.949 |
| CHOL | RCS10-CR | RCS10 | 0.687 | 0.639 | 0.394 |
|  | RCS10-CR | SWR/J | **0.026** | 0.120 | 0.066 |
|  | RCS10 | SWR/J | 0.099 | 0.246 | 0.236 |
| PPL | RCS10-CR | RCS10 | 0.272 | 0.208 | 0.120 |
|  | RCS10-CR | SWR/J | 0.056 | 0.194 | 0.120 |
|  | RCS10 | SWR/J | 0.492 | 0.633 | 0.781 |

^†^ One-way ANOVA with equal variance assumption with Tukey’s post-hoc tests. Bold text indicates *p*<0.05.

^‡^ One-way ANOVA with unequal variance assumption with Games Howell post-hoc tests. Bold text indicates *p*<0.05.

^§^ Kruskal-Wallis Test: Non-parametric tests with Dunn’s post-hoc tests. Bold text indicates *p*<0.05.

^¶^ Abbreviations: BW, body weight; FBG, fasting blood glucose; NEFA, non-esterified fatty acid; CHOL, cholesterol; TG, triglyceride; PPL, phospholipid; MNCV, motor nerve conduction velocity; SNCV, sensory nerve conduction velocity; IENFD, intraepidermal nerve fiber density.

**Table S4. Sensitivity analysis for data presented in Figure 4 and Supplemental Figures 1 and 4**

| **BL6 vs. RCS10** | | | | |
| --- | --- | --- | --- | --- |
| Variable | Weeks or min | Contrast | Parametric RM ANOVA | RM ANOVA with Robust SE |
| Longitudinal BW^†^ (weeks) | 10 | BL6 – RCS10 | **<0.001** | **<0.001** |
|  | 14 | BL6 – RCS10 | **<0.001** | **<0.001** |
|  | 16 | BL6 – RCS10 | **<0.001** | **<0.001** |
|  | 18 | BL6 – RCS10 | **<0.001** | **<0.001** |
|  | 20 | BL6 – RCS10 | **<0.001** | **<0.001** |
|  | 22 | BL6 – RCS10 | **<0.001** | **<0.001** |
|  | 24 | BL6 – RCS10 | **<0.001** | **<0.001** |
| Longitudinal FBG (weeks) | 10 | BL6 – RCS10 | **0.013** | **<0.001** |
|  | 14 | BL6 – RCS10 | 0.275 | 0.170 |
|  | 16 | BL6 – RCS10 | **<0.001** | **0.001** |
|  | 18 | BL6 – RCS10 | **0.002** | **0.001** |
|  | 20 | BL6 – RCS10 | **<0.001** | **<0.001** |
|  | 22 | BL6 – RCS10 | **<0.001** | **<0.001** |
|  | 24 | BL6 – RCS10 | **0.004** | **<0.001** |
| **SWR/J vs. TH** | | | | |
| Variable | Weeks or min | Contrast | Parametric RM ANOVA | RM ANOVA with Robust SE |
| Longitudinal BW (weeks) | 15 | SWR/J - TH | **<0.001** | **<0.001** |
|  | 17 | SWR/J - TH | **<0.001** | **<0.001** |
|  | 19 | SWR/J - TH | **<0.001** | **<0.001** |
|  | 21 | SWR/J - TH | **<0.001** | **0.001** |
|  | 23 | SWR/J - TH | **0.002** | **0.006** |
| Longitudinal FBG (weeks) | 15 | SWR/J - TH | **0.001** | **<0.001** |
|  | 17 | SWR/J - TH | **<0.001** | **<0.001** |
|  | 19 | SWR/J - TH | **<0.001** | **<0.001** |
|  | 21 | SWR/J - TH | **<0.001** | **<0.001** |
|  | 23 | SWR/J - TH | **<0.001** | **0.002** |
| **SWR/J vs. RCS10 vs. RCS10-CR** | | | | |
| Variable | Weeks or min | Contrast | Parametric RM ANOVA | RM ANOVA with Robust SE |
| Terminal GTT (min) | 0 | RCS10-CR -RCS10 | **0.035** | **<0.001** |
|  |  | RCS10-CR – SWR/J | 0.994 | 0.867 |
|  |  | RCS10 – SWR/J | 0.064 | **<0.001** |
|  | 15 | RCS10-CR -RCS10 | **0.001** | **<0.001** |
|  |  | RCS10-CR – SWR/J | 0.853 | 0.708 |
|  |  | RCS10 – SWR/J | **0.002** | **<0.001** |
|  | 30 | RCS10-CR -RCS10 | **<0.001** | **<0.001** |
|  |  | RCS10-CR – SWR/J | 0.790 | 0.446 |
|  |  | RCS10 – SWR/J | **<0.001** | **<0.001** |
|  | 60 | RCS10-CR -RCS10 | **<0.001** | **<0.001** |
|  |  | RCS10-CR – SWR/J | 0.902 | 0.768 |
|  |  | RCS10 – SWR/J | **<0.001** | **0.002** |
|  | 120 | RCS10-CR -RCS10 | **0.002** | **0.009** |
|  |  | RCS10-CR – SWR/J | 0.896 | 0.214 |
|  |  | RCS10 – SWR/J | **0.031** | **0.023** |
| Longitudinal BW (weeks) | 10 | RCS10-CR -RCS10 | 0.297 | 0.240 |
|  |  | RCS10-CR – SWR/J | **<0.001** | **<0.001** |
|  |  | RCS10 – SWR/J | **0.004** | **<0.001** |
|  | 12 | RCS10-CR -RCS10 | 0.101 | **0.007** |
|  |  | RCS10-CR – SWR/J | **<0.001** | **<0.001** |
|  |  | RCS10 – SWR/J | **0.004** | **<0.001** |
|  | 14 | RCS10-CR -RCS10 | 0.069 | **0.021** |
|  |  | RCS10-CR – SWR/J | **<0.001** | **<0.001** |
|  |  | RCS10 – SWR/J | **0.015** | **<0.001** |
|  | 16 | RCS10-CR -RCS10 | **0.036** | 0.101 |
|  |  | RCS10-CR – SWR/J | **<0.001** | **<0.001** |
|  |  | RCS10 – SWR/J | **0.016** | **0.019** |
|  | 18 | RCS10-CR -RCS10 | **0.022** | 0.077 |
|  |  | RCS10-CR – SWR/J | 0.367 | **0.044** |
|  |  | RCS10 – SWR/J | **0.002** | **0.003** |
|  | 20 | RCS10-CR -RCS10 | **<0.001** | **0.003** |
|  |  | RCS10-CR – SWR/J | >0.999 | 0.999 |
|  |  | RCS10 – SWR/J | **0.005** | **0.004** |
|  | 22 | RCS10-CR -RCS10 | **<0.001** | **0.002** |
|  |  | RCS10-CR – SWR/J | 0.755 | 0.302 |
|  |  | RCS10 – SWR/J | **0.014** | **0.018** |
|  | 24 | RCS10-CR -RCS10 | **<0.001** | **0.013** |
|  |  | RCS10-CR – SWR/J | 0.606 | 0.168 |
|  |  | RCS10 – SWR/J | **0.037** | 0.093 |
| Baseline GTT (min) | 0 | RCS10 – SWR/J | 0.124 | **<0.001** |
|  | 15 | RCS10 – SWR/J | **<0.001** | **<0.001** |
|  | 30 | RCS10 – SWR/J | **<0.001** | **<0.001** |
|  | 60 | RCS10 – SWR/J | **<0.001** | **<0.001** |
|  | 120 | RCS10 – SWR/J | **<0.001** | **<0.001** |
| Longitudinal FBG (weeks) | 10 | RCS10-CR -RCS10 | 0.997 | 0.998 |
|  |  | RCS10-CR – SWR/J | 0.120 | 0.165 |
|  |  | RCS10 – SWR/J | 0.137 | 0.073 |
|  | 12 | RCS10-CR -RCS10 | 0.745 | 0.811 |
|  |  | RCS10-CR – SWR/J | **<0.001** | **<0.001** |
|  |  | RCS10 – SWR/J | **0.003** | **<0.001** |
|  | 14 | RCS10-CR -RCS10 | 0.947 | 0.981 |
|  |  | RCS10-CR – SWR/J | 0.221 | 0.144 |
|  |  | RCS10 – SWR/J | 0.157 | 0.355 |
|  | 16 | RCS10-CR -RCS10 | 0.542 | 0.200 |
|  |  | RCS10-CR – SWR/J | **<0.001** | **<0.001** |
|  |  | RCS10 – SWR/J | **0.012** | **<0.001** |
|  | 18 | RCS10-CR -RCS10 | **0.042** | **0.011** |
|  |  | RCS10-CR – SWR/J | 0.659 | 0.323 |
|  |  | RCS10 – SWR/J | 0.443 | 0.324 |
|  | 20 | RCS10-CR -RCS10 | **0.040** | 0.081 |
|  |  | RCS10-CR – SWR/J | 0.973 | 0.819 |
|  |  | RCS10 – SWR/J | 0.147 | 0.125 |
|  | 22 | RCS10-CR -RCS10 | **<0.001** | **<0.001** |
|  |  | RCS10-CR – SWR/J | 0.977 | 0.865 |
|  |  | RCS10 – SWR/J | **<0.001** | **<0.001** |
|  | 24 | RCS10-CR -RCS10 | 0.869 | 0.850 |
|  |  | RCS10-CR – SWR/J | 0.747 | 0.604 |
|  |  | RCS10 – SWR/J | 0.952 | 0.933 |

^†^ Abbreviations: BW, body weight; FBG, fasting blood glucose; GTT, glucose tolerance test; min, minutes.

^‡^ Bold text indicates *p*<0.05.
